# Community-associated Methicillin-resistant *Staphylococcus aureus *Bacteremia and Endocarditis among HIV Patients: A cohort study

**DOI:** 10.1186/1471-2334-11-298

**Published:** 2011-10-31

**Authors:** Jon P Furuno, Jennifer K Johnson, Marin L Schweizer, Anayochukwu Uche, Oscar C Stine, Simone M Shurland, Graeme N Forrest

**Affiliations:** 1 Department of Pharmacy Practice, Oregon State University/Oregon Health and Sciences College of Pharmacy, Portland, OR, USA; 2 Department of Pathology, University of Maryland School of Medicine, Baltimore, MD, USA; 3 Department of Internal Medicine, University of Iowa Carver College of Medicine, Iowa City, IA, USA; 4 Oro Valley Hospital, Oro Valley, AZ, USA; 5 Department of Epidemiology and Public Health, University of Maryland School of Medicine, Baltimore, MD, USA; 6 Food and Drug Administration, Silver Spring, MD, USA; 7 Division of Infectious Diseases, Portland VA Medical Center and Oregon Health Science University, Portland, OR, USA

## Abstract

**Background:**

HIV patients are at increased risk of development of infections and infection-associated poor health outcomes. We aimed to 1) assess the prevalence of USA300 community-associated methicillin-resistant *Staphylococcus aureus *(CA-MRSA) among HIV-infected patients with *S. aureus *bloodstream infections and. 2) determine risk factors for infective endocarditis and in-hospital mortality among patients in this population.

**Methods:**

All adult HIV-infected patients with documented *S. aureus *bacteremia admitted to the University of Maryland Medical Center between January 1, 2003 and December 31, 2005 were included. CA-MRSA was defined as a USA300 MRSA isolate with the MBQBLO spa-type motif and positive for both the arginine catabolic mobile element and Panton-Valentin Leukocidin. Risk factors for *S. aureus*-associated infective endocarditis and mortality were determined using logistic regression to calculate odds ratios (OR) and 95% confidence intervals (CI). Potential risk factors included demographic variables, comorbid illnesses, and intravenous drug use.

**Results:**

Among 131 episodes of *S. aureus *bacteremia, 85 (66%) were MRSA of which 47 (54%) were CA-MRSA. Sixty-three patients (48%) developed endocarditis and 10 patients (8%) died in the hospital on the index admission Patients with CA-MRSA were significantly more likely to develop endocarditis (OR = 2.73, 95% CI = 1.30, 5.71). No other variables including comorbid conditions, current receipt of antiretroviral therapy, pre-culture severity of illness, or CD4 count were significantly associated with endocarditis and none were associated with in-hospital mortality.

**Conclusions:**

CA-MRSA was significantly associated with an increased incidence of endocarditis in this cohort of HIV patients with MRSA bacteremia. In populations such as these, in which the prevalence of intravenous drug use and probability of endocarditis are both high, efforts must be made for early detection, which may improve treatment outcomes.

## Background

*Staphylococcus aureus *bacteremia is a serious health condition associated with considerable morbidity and mortality among infected patients [1]. Negative outcomes associated with *S. aureus *bacteremia include prolonged hospital stay, endocarditis, sepsis, and death [1,2]. The changing epidemiology of *S. aureus*, including the increasing incidence and prevalence of methicillin-resistant *S. aureus *(MRSA) and the emergence of community-associated MRSA (CA-MRSA) further challenges clinicians when treating *S. aureus *bacteremia. A recent meta-analysis suggested that patients with MRSA bacteremia are at increased risk of death, and a longer post infection hospital length of stay compared to patients with bacteremia due to methicillin-susceptible *S. aureus *[1]. Furthermore, increasing evidence suggests that transmission of CA-MRSA is becoming more prevalent in healthcare settings and an emerging epidemic throughout the HIV community [3,4].

Decreased host immunity among HIV-infected patients places them at increased risk of infection including *S. aureus *bacteremia and associated poor outcomes [3,5,6]. Studies suggest that HIV patients are also at increased risk of CA-MRSA infections from overlapping community networks as well as the high prevalence of intravenous drug use in some areas [7,8]. Independent factors described as risks for MRSA bacteremia within HIV-infected patients have been intravenous drug use, hemodialysis and CD4 counts < 200 [9] while clinical outcomes suggest that incidence of re-infection and one-year mortality are high [3]. Recent data from a study by Kempker et al. suggested that CA-MRSA bacteremia was associated with advanced age, black race and AIDS infection as well as increased mortality compared to other strains [10]. However, despite this recent work, outcomes for CA-MRSA bacteremia and endocarditis among HIV patients have not been well-described. In this study we aimed to 1) assess the prevalence of USA300 community-associated methicillin-resistant *Staphylococcus aureus *(CA-MRSA) among HIV-infected patients with *S. aureus *bloodstream infections and 2) determine risk factors for infective endocarditis and in-hospital mortality among patients in this population.

## Methods

### Study Population

Prior to study commencement, the University of Maryland, Baltimore Institutional Review Board approved this study. This was a retrospective cohort study of all adult (age ≥18) HIV-infected patients with MRSA bacteremia admitted to the University of Maryland Medical Center (UMMC) between January 1, 2003 and December 31, 2005. During the study period, UMMC was a 648-bed, tertiary-care facility in Baltimore, MD, which included a 40-bed in-patient HIV service. Baltimore has the fifth highest incidence case report rate of people living with HIV/AIDS of any major metropolitan area in the U.S. with 2037.8 cases/100,000 population as of 2007and a high incidence of CA-MRSA at approximately 119 cases/100,000 population [11,12]. Data on rates of MRSA infection admitted to UMMC within this time period of being consistently around 50% have previously been published [13].

**Figure 1:**
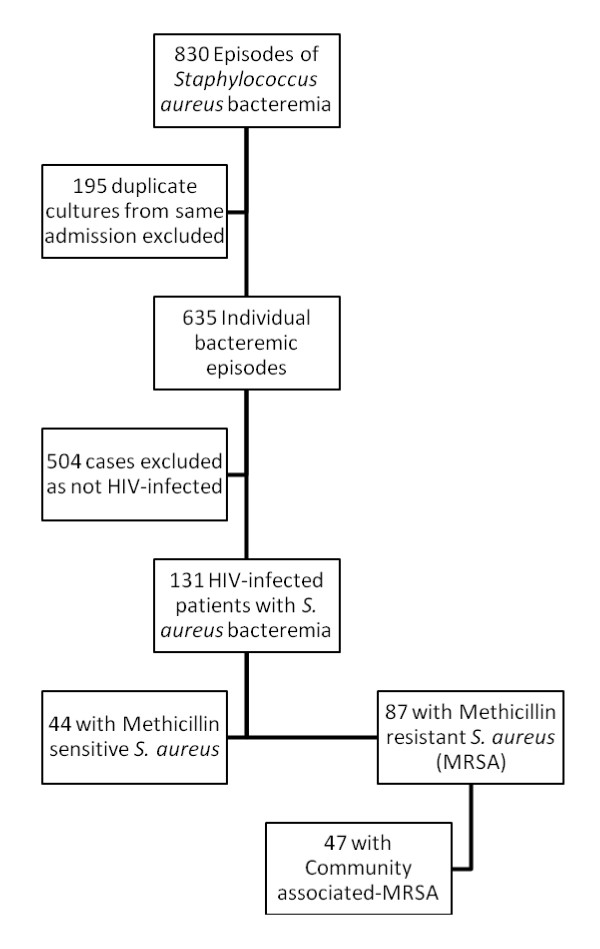
**Selection of HIV-infected patients with methicillin resistant *Staphylococcus aureus *bacteremia flowsheet**.

Patients with MRSA bacteremia for the time period evaluated were identified through Premier Safety Surveillor^® ^(Premier, Inc., Charlotte, N.C), an automated epidemiological surveillance program used by UMMC for infection control purposes and from which work has previously been described [14,15]. Duplicate cultures from the same admission were then removed and HIV status was then cross-referenced with the central data repository. The central data repository has been used in numerous epidemiologic studies of antimicrobial-resistant bacteria published in the peer-reviewed journals [16-18]. The flow diagram shows how the final patient population was determined. (Figure 1)

### Data Collection

Patient data were retrospectively collected via review of patient medical charts and through a central data repository of patients' administrative, pharmaceutical, and laboratory data. Chart review was performed by a clinician using a standardized data abstraction form. Data collected included past and current medical history: demographics, comorbid conditions, and clinical presentation on admission and just prior to positive blood culture. Clinical presentation included CD4 count (cells/mm*^3^*), and whether the patient was receiving effective antiretroviral therapy (ART) of at least 3 active agents. In addition, data on HIV viral load were collected if available within 6 weeks (on either side) of the admission.

### Variable Definitions

AIDS was defined as a CD4 < 200 cells/mm^3 ^or current opportunistic infection, Hepatitis C infection was defined as having documented serologic positivity, diabetes mellitus was defined as receiving insulin or oral hypoglycemic medication, hypertension was any patient receiving antihypertensive therapy, coronary artery disease was defined as any prior myocardial infarction, interventional procedure or bypass surgery. Illicit substance use (ISU) was determined by history and when possible toxicology screen results. End stage renal disease was defined as per National Kidney Foundation guidelines as stage 4 or 5 kidney disease including the need for renal replacement therapy [19]. Severity of illness was defined using the Acute Physiology and Chronic Health Evaluation (APACHE) II score at the time of initial positive blood culture collection. Endocarditis was diagnosed using either transesophageal (TEE) or transthoracic echocardiography (TTE) and defined according to the modified Duke criteria, with allocation to left-sided, right-sided, or bilateral disease [20]. Mortality was defined as in-hospital mortality on the index admission.

### Laboratory Methods

Blood cultures were drawn as per standard hospital policy from two separate sites and collected in blood culture bottles (BacTAlert, bioMerieux, Durham, NC). Cultures were placed in a continuous automated detection incubator. Positive blood culture bottles were plated onto standard growth media as per standard laboratory protocol and susceptibilities were performed using disk diffusion following the Clinical and Laboratory Standards Institute (CLSI) guidelines [21,22]. The first *S. aureus *positive blood culture per patient per admission was included in the analysis. Thus, patients could have contributed more than one episode of *S. aureus *bacteremia to the data, so long as the cultures occurred on different inpatient admissions and were not treated for endocarditis in last 90 days.

MRSA *spa*-type was determined using the methods proposed by Shopsin et al. and Harmsen et al [23,24]. Presence of Panton-Valentine leukocidin (PVL) and arginine catabolic mobile element (ACME) was determined using the previously described protocols [25,26]. CA-MRSA was defined as a USA300 MRSA isolate with the MBQBLO spa-type motif that was both PVL and ACME positive as has been previously described [25,27,28]. This method was validated by performing pulsed-field gel electrophoresis on isolates that met two of the three following criteria: MBQBLO spa-type motif, PVL positive, ACME positive. Of those that met two of the three criteria, none were USA300. Although there are other CA-MRSA strains, USA300 MRSA is the predominant strain type associated with CA-MRSA colonization and infection [29].

### Statistical analysis

Student's t-tests, chi-square, Fisher's exact, and Wilcoxon rank sum tests were used to identify differences between patients with an without CA-MRSA and between those who and did and did not develop endocarditis or die on the index admission. All variables that were statistically significant (α = 0.1) in the bivariable analyses were included in the initial (full) multivariable logistic regression model. In each of the multivariable analyses performed, variables not significantly associated (α = 0.05) with the outcome were removed from the model. Each of the removed variables was then re-inserted into the model to assess if its presence altered the regression coefficient by 20% or more. If so, this confounding variable was included in the final model. The resulting multivariable logistic regression model was considered the final model and was used to calculate odds ratios (ORs) and 95% confidence intervals (CI) for the remaining risk factors. All analyses were conducted using SAS statistical software, Version 9.1.2 (SAS Institute, Cary, NC).

## Results

We identified 131 individual episodes of *S. aureus *bacteremia among HIV-positive patients during the study period. All episodes occurred within 72 hours of admission. Characteristics of the study population are displayed in Table 1.Twenty one percent of the patients had a diagnosis of AIDS. The mean CD4 count of the cohort was 126 cells/per mm^3 ^(standard deviation =133 cells/per mm^3^) and median CD4 count was 56 cells/per mm^3 ^(interquartile range [IQR] 16-169 cells/per mm^3^). The mean HIV viral load was 225,000 copies/ml and only 15 patients having a suppressed HIV viral load in this cohort. Of note, prevalence of co-infection with hepatitis C virus was 63%, prevalence of ISU was 50%, and 30% of patients were receiving ART. Approximately 66% (n = 87) of *S. aureus *isolates were MRSA of which 47 (54%) were CA-MRSA. Antibiotic susceptibilities and molecular characteristics of the *S. aureus *isolates are displayed in Table 2. Initial empiric antibiotic selection for the treatment of CA-MRSA bacteremia would not be effective therapy in 27 of the patients (31%).

**Table 1:** Characteristics of the Patients with *Staphylococcus aureus *bacteremia (n = 131)

Characteristic	n (%)
**Mean (SD) Age, (years)**	41.5 (7.5)

**Male Sex**	83 (63.4)

**African American**	119 (91)

**Receiving ART**	39 (29.8)

**Mean (SD) APACHE Score**	12.1 (5.3)

**CD4 <200 cells/mm*^3^***	28 (21.4)

**Hepatitis C**	82 (62.6)

**Diabetes Mellitus**	3 (2.3)

**Coronary Artery Disease**	0 (-)

**Hypertension**	13 (9.9)

**Intravenous Drug Use**	66 (50.4)

**End-stage Renal Disease**	24 (18.3)

**Median (IQR) Length of Stay (days)**	7 (5, 14)

**Endocarditis**	63 (48.1)

**Right-sided only**	51 (38.9)

**Left-sided only**	8 (6.1)

**Bilateral**	4 (3.1)

**In-hospital Mortality**	10 (7.6)

Note: ART: Antiretroviral therapy, SD: standard deviation, IQR: interquartile range, APACHE: Acute Physiology and Chronic Health Evaluation score.

**Table 2:** Characteristics of the *Staphylococcus aureus *isolates (n = 131)

Characteristic	n (%)
**Antibiotic Susceptibilities**	

**Methicillin Resistant**	87 (66)

**Clindamycin Resistant**	25 (19.1)

**Trimethoprim-Sulfamethoxazole Resistant**	42 (32.1)

Molecular Characteristics*	

Panton Valentine Leukocidin (PVL)*	53 (61.6)

Arginine Catabolic Mobile Element (ACME)*	50 (58.1)

USA300 MRSA*	47 (54.0)

*Proportion is only among MRSA isolates

The prevalence of endocarditis was approximately 48% and in-hospital mortality during the index admission was approximately 8%. Among the 63 patients with endocarditis, 51 (81%) had right-sided endocarditis only. Of the 31 MRSA patients (35%) that had both a TEE and TTE, 5 vegetations (2 aortic, 2 mitral, 1 pulmonic) were detected by TEE that were not seen by TTE, but this was not related to whether they had CA or Non-CA-MRSA infection. Metastatic MRSA infections were common with septic pulmonary emboli occurring in 11 of the CA-MRSA patients and 6 non-CA-MRSA patients (p = 0.32). However, there were significantly more deep abscesses in the CA-MRSA group compared to the non-CA-MRSA group (17 versus 1 p < 0.001). Of the 17 CA-MRSA patients with deep abscesses, 7 patients had lung abscess or empyema, 5 patients had pyomyositis (psoas and spinal muscle abscesses), 2 patients had renal abscesses, 2 patients had a brain abscess and one patient had a pericardial empyema. The non-CA-MRSA group deep abscesses had one epidural abscess. The mean duration of bacteremia was not significantly different between the patients with CA-MRSA (mean 1.85 days) and those with non-CA-MRSA (1.45 days) p = 0.43).

Table 3 displays the unadjusted odds ratios for the association between different variables and endocarditis. Patients with CA-MRSA infections had nearly three times the odds of endocarditis and this association was statistically significant (OR = 2.73, 95% CI = 1.30, 5.71). No other variables were significantly associated with endocarditis in the bivariate analyses. Logistic regression was use to perform multivariable analyses, but no other variables were significantly associated with endocarditis. No variables were significantly associated with in-hospital mortality. CA-MRSA was associated with nearly three times the odds of in-hospital mortality, (OR = 2.93, 95% CI = 0.78, 10.96). We could not fully evaluate the associations between APACHE ≥ 11 and receiving ART and mortality, because none of the patients who received ART died and none of the patients with APACHE less than 11 died.

**Table 3:** Odds Ratios (ORs) and 95% Confidence Intervals (CIs) for Associations of various Risk Factors for Endocarditis and In-hospital Mortality among HIV Patients with *Staphylococcus aureus *Bacteremia (n = 131)

	Endocarditis	In-hospital Mortality
**Characteristic**	OR (95% CI)	OR (95% CI)

**CA-MRSA**	2.73 (1.30, 5.71)	2.93 (0.78, 10.96)

**Age**	0.98 (0.94, 1.03)	1.08 (0.99, 1.17)

**Male Sex**	0.46 (0.22, 0.94)	0.35 (0.10, 1.33)

**Receiving ART**	0.67 (0.31, 1.42)	*

**APACHE II ≥11**	1.71 (0.85, 3.42)	**

**CD4 <200 cells/mm^3^**	2.01 (0.87, 4.62)	0.47 (0.08, 2.91)

**Hepatitis C**	1.23 (0.60, 2.50)	0.57 (0.16, 2.08)

**Hypertension**	0.92 (0.40, 2.12)	0.91 (0.18, 4.57)

**Intravenous Drug Use**	1.79 (0.90, 3.59)	1.58 (0.42, 5.87)

**End-stage Renal Disease**	1.23 (0.48, 3.12)	0.56 (0.07, 4.68)

Note: CA-MRSA: community-associated methicillin-resistant *Staphylococcus aureus*, ART: antiretroviral therapy, APACHE: Acute Physiology and Chronic Health Evaluation score

*Could not be calculated because no patients receiving ART died.

**Could not be calculated because no patients with APACHE <11 died.

## Discussion

We present the largest cohort of HIV patients with CA-MRSA bacteremia and endocarditis documented to date. These data suggest a high prevalence of CA-MRSA among HIV patients presenting with *S. aureus *bacteremia at our institution and that CA-MRSA was significantly associated with the development of endocarditis compared to non-CA-MRSA. Furthermore, although not significant, patients with CA-MRSA also appeared to be at increased risk of death compared to patients with other *S. aureus *bloodstream infections.

Kempker et al. presented a large cohort of CA-MRSA bacteremia cases from eight hospitals over 3 years in the Atlanta, GA metropolitan area.(10) They had 414 episodes of CA-MRSA bacteremia of which 63 (16.2%) occurred in HIV positive patients and only 17 (4.1%) were associated with endocarditis. They observed greater in-hospital mortality (17%) compared to 8% in our study, however similar to the patients in our cohort, they observed more deep abscesses in their CA-MRSA group compared to the non-CA-MRSA group [10]. Wang et al. performed a study of adult patients with *S. aureus *bacteremia in Taiwan, however, only 3 (10%) of their CA-MRSA bacteremia episodes had endocarditis, and none were HIV positive. On their univariate analysis, women appear to have a two-fold increase of dying with CA-MRSA bacteremia, a similar finding to our study [30]. Several other papers on CA-MRSA bacteremia have had very few cases of endocarditis most of which appear to be associated with deep abscesses and pulmonary disease [31-34]. Kreisel et al evaluated CA-MRSA bacteremia and severe sepsis at 4 Veteran Affairs (VA) centers, which includes the Baltimore VA medical center and demonstrated that CA-MRSA was associated with more severe sepsis (adjusted relative risk 1.82, confidence intervals 1.16-2.87, p = 0.01) [35]. They also noted that there was a greater propensity to ISU and African American patients having these severe infections, which is consistent with our population [35,36].

Another major difference between our study and previously published work is the antibiotic susceptibility profile of our CA-MRSA strains compared to other studies with high levels of trimethoprim-sulfamethoxazole (TMP-STX) resistance, which was 32% of all isolates and 48% of the CA-MRSA isolates. This compares to ranges of 0-13% in other published studies [30,32-34]. TMP-STX resistance could have several possible explanations given the patient population examined; 1) As the majority of the patients had a history of intravenous drug usage, it is possible they had more abscesses in the past and had been treated with TMP-STX several times or 2) the majority of the patients had CD4 < 200 cells/mm^3 ^and were most likely taking TMP-STX for prevention of pneumocystis pneumonia. The second explanation appears more likely as our clindamycin resistance was similar to other studies [30,32-34].

Our data compare similarly to data published from Detroit, which also has high rates of CA-MRSA. CA-MRSA was responsible for 35% of bloodstream infections in their series, but there were only 3% HIV infected patients in their cohort [37]. Lalani et al. during a multicenter clinical trial that the reported a prevalence of CA-MRSA was 12% among 230 *S. aureus *isolates and 31% among the 88 typed MRSA isolates in a multicenter clinical trial.(28) Incidence of endocarditis was 23%. However, in contrast to our study, the Lalani et al. study population had a low prevalence of HIV/AIDS of approximately 3% and thus a thorough comparison to the patients in our study is not possible. Gebo et al. assessed risk factors and outcomes of 58 cases of infective endocarditis among HIV-infected patients and identified intravenous drug use, low (<50 cells/mm^3^) CD4 counts, and high (HIV RNA >100,000 copies/ML) viral load as independent risk factors for endocarditis. (3) Although *S. aureus *was the most prevalent organism (69% of cases) in their study, only 28% of *S. aureus *isolates were MRSA, which also complicates comparison to our data, which focused on differences in risk factors and outcomes between CA-MRSA and non-CA-MRSA.

We observed relatively low mortality (~8%) despite that greater than 20% of patients had a CD4 count of <200 cells/mm^3 ^and less than 30% were receiving ART. The low rate of ART use in our population with high ISU reflects the difficulty in obtaining compliance in this population. There have been several studies on the difficulties in getting the HIV infected ISU patients to establish and maintain antiretroviral usage in the city of Baltimore. It has been suggested that ISU infected patients with HIV who were not receiving ART tended to be active drug users without clinical disease [38]. It may explain why there was higher mortality in women in our study with the sex for illicit drug trade delaying the presentation for care [39].

Our results contrast previous studies, which observed mortality from 18% to 52% [3,5]. However, the associations between ISU, right-sided endocarditis and low relative mortality have been previously described, even among HIV patients [40]. Recall that more than 80% of our patients with endocarditis had right-sided disease only. Furthermore, as has been previously reported, the criteria used to measure mortality often vary in studies of infectious disease outcomes [41]. For example, the 52% mortality reported by Gebo et al. included all deaths within one year, some of which were likely not attributable to the endocarditis episode [3]. The low mortality in this study relative to the potential seriousness of the underlying infection, suggests that although CA-MRSA was significantly associated with endocarditis, this may be more related to risk associated with intravenous drug use and other outcomes such as mortality may still not be as severe as among other endocarditis patients [42,43]. Furthermore, other studies have suggested that HIV infection was not significantly associated with mortality among patients with infective endocarditis in which 65% were caused by *S. aureus*, 38% of patients were HIV-positive and 16% mortality was observed [6].

The retrospective nature of the study limited which data were able to be collected and the availability of *S. aureus *isolates. Furthermore, we used a molecular definition of USA300 MRSA to define CA-MRSA. While USA300 remains the predominant CA-MRSA, we likely underestimated the prevalence of CA-MRSA in this study. Lastly, given the high prevalence of CA-MRSA, ISU, and HIV in the study population and in Baltimore City in general, the generalizability of these data to other patient populations outside of urban, tertiary-care settings should be performed with caution.

## Conclusions

In conclusion, CA-MRSA was significantly associated with an increased prevalence of endocarditis in this cohort of HIV patients with MRSA bacteremia. Despite that we observed lower mortality compared with other studies, these patients remain at considerably higher risk for the development of endocarditis and related mortality. In populations such as these, in which the prevalence of intravenous drug use and probability of endocarditis are both high, efforts must be made for early detection, which may improve treatment outcomes.

## Competing interests

In the past 5 years J.P.F. and M.L.S. have served as consults for Cubist Pharmaceuticals, Inc. Also, G.N.F. has received research funding and honoraria from Cubist Pharmaceuticals, Inc. and Pfizer Inc. M.L.S. has received research funding from Pfizer, Inc.

## Authors' contributions

AU and GF conceived the study, participated in the design and data collection and drafted the final manuscript. JF participated in study design, obtaining grant support and drafted the final manuscript. MLS and SMS participated in the statistical analysis and were involved in drafting the final manuscript. JKK and OCS performed the molecular testing on the clinical specimens and contributed in drafting the manuscript.

All authors read and approved the final manuscript.

## Pre-publication history

The pre-publication history for this paper can be accessed here:

http://www.biomedcentral.com/1471-2334/11/298/prepub
